# Bilateral Nonneoplastic Heterotopic Submandibular Glands Discovered Postmortem: A Case Study

**DOI:** 10.1155/crot/8997735

**Published:** 2026-06-23

**Authors:** Sydney M. McSweeney, Bryan E. Burk, Krista L. Denning, Maria A. Serrat

**Affiliations:** ^1^ Marshall University Joan C. Edwards School of Medicine, Huntington, West Virginia, USA, marshall.edu; ^2^ Department of Biomedical Sciences, Marshall University Joan C. Edwards School of Medicine, Huntington, West Virginia, USA, marshall.edu; ^3^ Marshall Health Department of Pathology, Marshall University Joan C. Edwards School of Medicine, Huntington, West Virginia, USA, marshall.edu

## Abstract

The submandibular glands are salivary glands located under the mandible that secrete saliva into the oral cavity. The occurrence of heterotopic submandibular glands is an uncommon anatomical variation that is usually found unilaterally. There are few known reports of bilateral heterotopic submandibular glands; this paper describes a rare case of bilateral heterotopic submandibular glands that were discovered incidentally during routine dissection of a cadaver for medical education purposes and subsequent histological examination. The heterotopic glands were located in the carotid triangle at the level of the hyoid bone and appeared to receive blood supply from the facial artery and innervation from the facial nerve, similar to that of typical submandibular glands in normal anatomical location. Venous drainage was into the facial vein and communicating jugular vein. Dissection of each bilateral heterotopic submandibular gland revealed a duct oriented toward the oral cavity, suggesting that it was a functional salivary gland. Histological analysis of the glands and nearby deep cervical chain lymph nodes revealed normal salivary gland and lymph node structure, with no evidence of neoplasia. Encountering such anatomical variations during cadaveric dissection as part of medical education is an irreplaceable learning experience that increases the understanding of pathologies and clinical competency.

## 1. Introduction

### 1.1. Anatomy and Neurovasculature of Typical SMGs

The submandibular salivary glands (SMGs) are one pair of three main salivary glands which include the parotid, submandibular, and sublingual glands. The submandibular glands are second largest in size behind the parotid glands yet produce the most saliva—approximately 70% in the unstimulated state [1]. Typically, the SMGs are located in the posterior portion of the submandibular triangle which is created by the body of the mandible superiorly, the anterior belly of the digastric muscle medially, and the posterior belly of the digastric muscle laterally [1, 2]. The SMGs are also typically comprised of two lobes—superficial and deep—separated by the mylohyoid muscle. Additionally, excretion from the SMGs occurs mainly through Wharton’s duct which originates at the SMG and terminates in the oral cavity at the sublingual caruncula [1].

The SMGs are innervated by parasympathetic fibers from the facial nerve (cranial nerve VII) that travel toward the oral cavity via the chorda tympani. These fibers then synapse in the submandibular ganglion and extend onto the sublingual and submandibular glands. Meanwhile, sympathetic innervation to these glands is supplied by postganglionic fibers extending from cell bodies synapsing in the superior cervical ganglion [1, 3]. Unlike most of the body, the parasympathetic and sympathetic autonomic nervous systems work together in all three major salivary glands to stimulate the secretion of saliva [4]. The SMGs primarily receive their blood supply from the submental and sublingual arteries which are branches of the tortuous facial artery which originates from the external carotid artery. The common facial and sublingual veins drain the glands into the internal jugular vein [1].

### 1.2. Development

Development of SMGs is generally classified into five stages—prebud, initial bud, pseudoglandular, canalicular, and terminal bud [5]. During the prebud stage, the SMGs are first visible as a condensation of epithelial cells in the floor of the mouth next to the tongue. During the initial bud stage, the thickening parenchyma grows down into the first branchial arch mesenchyme and proliferates into a solid cord that terminates into a bulb [6–8]. Both of these stages occur at Carnegie stages (CS) 16 and 17, which correspond to approximately Week 6 of the developing embryo [8]. The pseudoglandular stage is characterized by repeated branching of the primordium of the parenchyma; this lobulation process occurs at CS 18‐19 (Week 7) and creates a bush‐like structure comprised of epithelial branches and terminal epithelial buds [6–8]. These branches and buds then hollow out via epithelial cell apoptosis to give rise to the ductal system and acini during the canalicular and terminal bud stages [6, 7]. It is hypothesized that the formation of an accessory/heterotopic SMG typically occurs around Week 7 of development as Wharton’s duct forms which may invaginate into two separate locations [9, 10].

### 1.3. Heterotopic Salivary Glands

Distinction in the literature between “accessory” and “heterotopic” submandibular glands is obscure, as the terms have been used interchangeably regardless of the presence of an excretory duct or orthotopic submandibular glands. Accessory SMGs would indicate the presence of both orthotopic SMGs and unilateral or bilateral glands in an ectopic position. Heterotopic SMGs would indicate a lack of orthotopic SMGs and only the presence of a unilateral or bilateral ectopic SMGs. Other cases of salivary gland tissue in the neck have been reported with the vast majority being found in patients presenting with a unilateral neck mass, cyst, nodule, or draining sinus [11, 12]. Most cases in the literature are found in children or young individuals with a unilateral branchial fistula exiting the anterior surface of the sternocleidomastoid muscle [12]. The presence of accessory and/or heterotopic salivary glands bilaterally is rare and has only been described a few times in the literature.

One case of bilateral submandibular glands was described by Codjambopoulo et al. in 1992 using the term “accessory.” This anomaly was found in a 47‐year‐old woman presenting for a persistent, painful swelling in the left mandible that had lasted for 4 weeks; the authors performed imaging using sialography and described a bilateral double formation of the submandibular duct with each duct having a separate ostium and draining distinct, noncommunicating glandular portions [13]. Another case was described by Owaja et al. in 2014 using the term “heterotopic” to describe bilateral salivary tissue in the anterior neck in a 24‐year‐old male presenting with intermittent clear drainage on both sides of the neck anterior to the sternocleidomastoid muscle. Fistulography was performed and revealed a sinus from the left fistula extending in the medial‐caudal direction; fistulography of the right side was not possible [14].

Few cases have been documented involving bilateral heterotopic SMGs found postmortem during cadaveric dissection. One case was found incidentally in an 86‐year‐old male cadaver during a neck dissection. These glands were found bilaterally and symmetrically around the level of the hyoid bone. The authors chose to classify the glands as “heterotopic” because they were encapsulated in their own fascia and drained via their own excretory duct; however, orthotopic SMGs were also present. Histopathological analysis was performed which showed predominantly serous with interspersed mucinous acini, characteristic of a typical SMG [15]. Perhaps, the most similar case to ours was described by Tassema in 2024 during the dissection of a 77‐year‐old male cadaver. These glands were found bilaterally within the carotid triangle without the presence of orthotopic SMGs. This case, however, described innervation by branches of the lingual nerve and did not describe a vascular supply or histopathological analysis [16].

## 2. Materials and Methods

A dissection of the head and neck of an 83‐year‐old male cadaver was performed as part of medical training and followed all institutional guidelines and Human Gift Registry regulations for the use of human material. Both the right and left heterotopic SMGs were collected from the cadaver along with several lymph nodes of the deep cervical chain and a lobule of adipose tissue containing vessels discovered under the left side of the mandible in the normal anatomical location of an orthotopic SMG. The tissue was microscopically analyzed using hematoxylin and eosin (H & E) staining techniques and underwent pathological analysis. This was done to correctly identify the tissue type and assess for neoplasia.

## 3. Results

**FIGURE 1:**
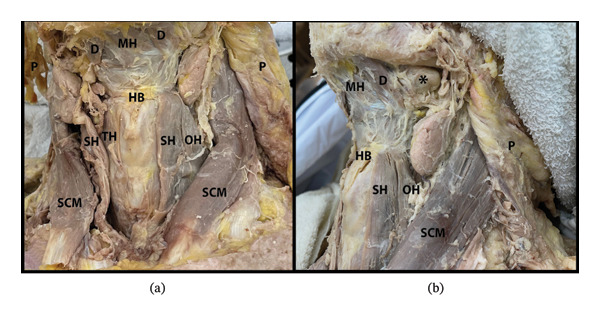
Viewing the neck anteriorly, with the platysma (P) reflected, there are bilateral heterotopic submandibular glands lying around the level of the hyoid bone (HB) (a). The left side of the neck shows a lobule of adipose tissue (∗) surrounding vasculature in the expected anatomical location of an orthotopic SMG (b). D: digastric muscle (anterior belly), HB: hyoid bone, MH: mylohyoid muscle, OH: omohyoid muscle (superior belly), P: platysma muscle, SCM: sternocleidomastoid muscle, SH: sternohyoid muscle, TH: thyrohyoid muscle.

Once the skin and adipose tissue were removed from the anterior neck and the platysma was reflected, two abnormal presumed glandular structures were discovered situated near the level of the hyoid bone bilaterally (Figure 1a). These glandular structures sit in the carotid triangle created by the posterior belly of the digastric muscle superiorly, the omohyoid muscle inferomedially, and the sternocleidomastoid laterally. The inferior one‐third of the glands lie at or below the level of the hyoid bone, while the superior two‐thirds of the glands lie above the level of the hyoid bone. The left side of the anterior neck also shows a lobule of fat in the expected anatomical location of an orthotopic SMG (Figure 1b). Upon closer inspection, the fat lobule encases end vasculature containing both arteries and veins. Therefore, orthotopic SMGs were not discovered in the posterior portion of the submandibular triangle on either side of the neck as would be expected.

Separating the presumed heterotopic SMGs from surrounding fascia and structures proved difficult as significant tissue matting was present, especially in the superolateral portion of the glands. This discovery, along with several enlarged lymph nodes of the deep cervical chain, supported the possibility of neoplastic origins and the need for histological analysis. However, the bilateral presentation and similar size of the glands in relation to each other were not in support of neoplastic origins. The following measurements were taken in the axial plane: the right heterotopic SMG measures approximately 1.80 cm from medial to lateral and 3 cm from superior to inferior; the left heterotopic SMG measures approximately 1.75 cm from medial to lateral and 3.25 cm from superior to inferior. Neither gland is uniform in size as there are lobular protrusions, especially on the superior and medial poles of the glands. Therefore, since the glands are not perfect ovals, these measurements are maximal and taken from the largest lobular protrusion.

**FIGURE 2:**
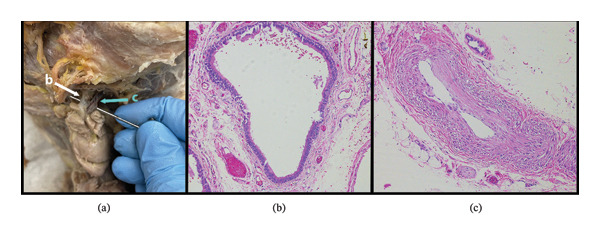
The white arrow corresponds to panel (b) of the figure which indicates an excretory salivary duct, while the cyan arrow corresponds to panel (c) of the figure which indicates a vein. Both structures are diving underneath the posterior belly of the digastric muscle (a) and are visualized at 100x with H & E stain.

**FIGURE 3:**
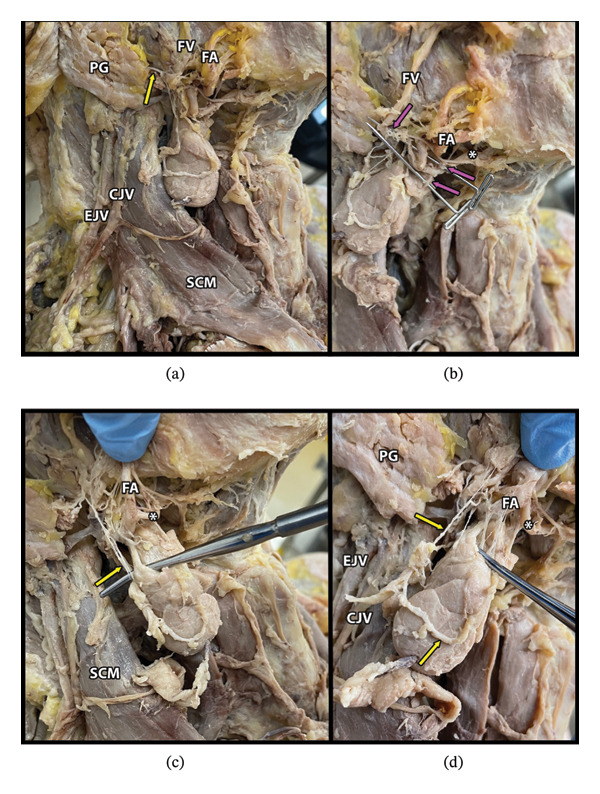
The heterotopic submandibular gland has venous drainage via a vein exiting the inferior pole of the gland and draining into the communicating jugular vein (CJV) as well as via a branch of the submental vein at the superior pole denoted by pink arrows (a, b). The heterotopic submandibular gland appears to receive arterial blood supply from a branch of the facial artery (FA) proximal to the takeoff of the submental artery, which is denoted as ∗ to maintain its visibility (c). The gland also appears to be innervated by the marginal mandibular branch of the facial nerve (CNVII MMB) with a branch entering the gland at both the superior pole and the middle anterior surface; the CNVII MMB is denoted by yellow arrows (a, c, d). CJV: communicating jugular vein, EJV: external jugular vein, FA: facial artery, FV: facial vein, PG: parotid gland, SCM: sternocleidomastoid muscle.

At the superior pole of the glands lies two tubular structures diving underneath the posterior belly of the digastric muscle (Figure 2a). The more superolateral structure is an excretory duct, presumably leading to the oral cavity to eject saliva, as confirmed by histology in Figure 2b. The more inferomedial structure is a vein (Figure 2c) that runs the length of the glands and exits at the inferior pole, draining into the communicating jugular vein (CJV) (Figure 3). Figure 3a and Figure 3c best illustrate this vein exiting the inferior pole of the right gland and looping around to drain into the CJV. The excretory duct and vein shown in Figure 2 were not further dissected in order to preserve the integrity of the cadaver for medical education. Additional venous drainage is located superomedially via a branch off the submental vein which entangles with the submental artery. The branch of the submental vein draining the superior part of the heterotopic SMG dips behind the submental artery; the submental vein then drains into the facial vein denoted by pink arrows (Figure 3b).

Arterial supply to the glands is provided by a single vessel entering the gland on its anterior surface about one‐third of the way inferiorly from its superior pole. This artery branches off the facial artery proximal to the takeoff of the submental artery (Figure 3c). This was the only arterial supply discovered during dissection. A marginal mandibular branch of the facial nerve (cranial nerve VII) was traced to the heterotopic SMG. CNVII MMB emerges from underneath the parotid gland and travels inferiorly to provide multiple branches that enter the glands at both the superior pole and anteromedial surface (Figure 3a,c,d). Much of the adipose tissue surrounding the nerve entering the anteromedial surface of the right gland was not removed so as not to jeopardize the integrity of the nerve.

**FIGURE 4:**
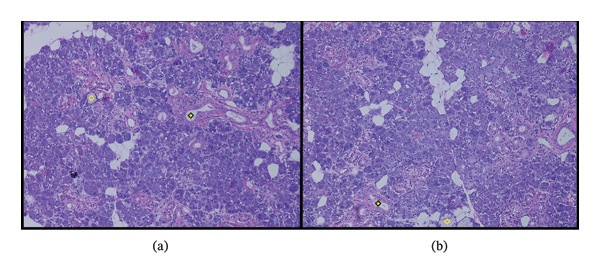
The left (a) and right (b) submandibular glands stained with H & E at 100x. Both slides show predominantly serous with interspersed mucinous acini (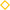). There is also normal adipose tissue infiltration. Ducts (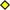) are normal in size and composition. There is no evidence of neoplasia.

**FIGURE 5:**
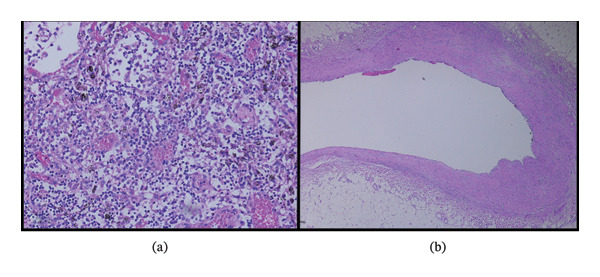
Normal lymph node tissue (a) at 200x with H & E shows nonneoplastic lymphoid tissue with interspersed anthracotic pigment. Normal vein (b) at 40x with H & E surrounded by adipose tissue. The sample in b was taken from the fat lobule containing end vasculature located in the normal anatomical position of an orthotopic SMG.

Histological analysis of both the left and right heterotopic SMGs reveals normal lobular architecture with predominantly basophilic serous acini with interspersed mucinous acini which appear unremarkable (Figure 4). The ducts are also normal in size and composition; adipose infiltration appears normal, and there is no evidence of neoplasia (Figure 4). Lymph nodes of the deep cervical chain were also harvested for histological analysis due to their large size. Microscopically, there is no evidence of neoplasia. There is interspersed anthracotic pigment present which could be due to a variety of environmental factors (Figure 5a). A portion of the lobule of fat observed in Figure 1b was excised to confirm the absence of orthotopic SMG tissue. Upon gross examination, the fat contained only end vasculature. Microscopic analysis confirmed this notion by the presence of normal veins and arteries surrounded by adipose tissue (Figure 5b).

## 4. Discussion

Heterotopic/accessory SMGs are an uncommon anomaly usually found unilaterally in patients presenting with an asymptomatic nodule or draining sinus in the anterior neck overlying the sternocleidomastoid muscle; bilateral discovery is rare. Considering the obscurity in the literature regarding the distinction between “accessory” and “heterotopic” salivary gland tissue, the authors have chosen to use “heterotopic” in this case due to the absence of orthotopic SMGs. In the case being presented, bilateral heterotopic SMGs were discovered postmortem in a cadaver during a head and neck dissection performed for medical education. These glands were discovered in the carotid triangle at the level of the hyoid bone with the majority of the neurovasculature being located at the superior pole.

In this case study, the bilateral heterotopic SMGs received their blood supply and drainage from branches of the facial artery and vein, respectively, similar to that of orthotopic SMGs. Branches of the sublingual artery and vein do not appear to contribute to these anomalous glands, likely due to their increased distance from the oral cavity. In addition to the facial vessels, there was an additional vein observed beginning from underneath the posterior belly of the digastric muscle, traveling through the length of the gland, and exiting its inferior pole to drain into the CJV. Innervation to these heterotopic SMGs appeared to be from a branch of the facial nerve (cranial nerve VII), similar to normal SMGs [1, 3]. The left side of the anterior neck also revealed a lobule of fat in the normal anatomical location of an orthotopic SMG and, upon excision and microscopic analysis, revealed end vasculature surrounded by adipose tissue.

While there is some variation in reported average size of the normal SMGs, the heterotopic SMGs discovered in this case were considerably larger than those reported in the literature [17, 18]. The histological analysis showed a basophilic array of normal glandular tissue with primarily serous acini and interspersed mucinous acini, which is characteristic of normal SMGs. The ducts and adipose tissue found within the glands were also unremarkable. These findings suggest that the heterotopic SMGs are nonneoplastic in origin. The lymph nodes analyzed from the deep cervical chain were unremarkable except for interspersed anthracotic pigment that could be consistent with tobacco use, coal dust, or other environmental factors [19]; however, the patient history is unknown.

Cadaveric dissection is an integral part of medical education as it provides the opportunity to not only learn gross anatomy but also encounter rare anatomical variations. These experiences are invaluable in both surgical and nonsurgical specialties alike and enhance understanding of disease pathologies and how they affect the body. Gross anatomy lab in medical education is so valuable that institutions, such as New York University, University of California at San Francisco (UCSF) and at Davis, the University of Hawaii, and the University of Washington, that once decided to eliminate cadaveric dissection from their curriculum, reversed their decisions quite quickly when it became apparent student understanding and clinical competency became compromised as a result. Even pedagogies such as optional lab dissections and prosection‐only teaching tested at the aforementioned universities proved inferior to small group cadaveric dissection [20].

In conclusion, this paper described a rare case of bilateral accessory SMGs that were identified during a cadaveric dissection for medical education. Gross anatomical and histological analyses suggest that the glands were not neoplastic in origin and likely arose anomalously during development. Gross anatomy lab as part of medical training is essential for understanding not only the expected anatomical location of structures but also for encountering anatomical variation and understanding its clinical implications.

## Funding

No funding was received for this manuscript.

## Consent

No written consent has been obtained from the patient(s) as there are no patient identifiable data included in this case report.

## Conflicts of Interest

The authors declare no conflicts of interest.

## Data Availability

The data that support the findings of this study are available on request from the corresponding author. The data are not publicly available due to privacy or ethical restrictions.
